# Establishing safety huddles on a general adult acute psychiatric ward: staff's views and relation with restrictive practice

**DOI:** 10.1192/bjo.2021.485

**Published:** 2021-06-18

**Authors:** Sidra Chaudhry, Nicoletta Lekka

**Affiliations:** Sheffield Health and Social Care NHS Foundation Trust

## Abstract

**Aims:**

To establish Safety Huddles (SH) on an acute general adult psychiatric ward, exploring links to restrictive practice. Additionally, to obtain multidisciplinary staff feedback on SH's impact on their workload/wellbeing and on patient care, and to identify barriers in implementation.

Background: A SH is a multidisciplinary daily briefing focused on patients most at risk, held at a fixed time and place, lasting max 5-10 minutes. Effective SH involve agreed actions, are informed by multidisciplinary staff feedback of data and provide the opportunity to appreciate and celebrate success in reducing harm. SH are a valuable team building activity, promoting situational awareness and helping with prioritising daily tasks.

**Method:**

SH were introduced on September 2020. Templates were developed to prompt staff how to facilitate. Staff were encouraged to identify key goals and reflect on issues in the last and next 24 hours. Each participant was allocated a role, e.g. record keeping or dissemination of information. In December 2020, records of incidence of restrictive practice (numbers of restraints, seclusions and rapid tranquilisations) were obtained for the periods June-August 2020 and September-November 2020. Additionally, staff feedback was obtained through a short anonymous Survey Monkey questionnaire. It explored whether SH had an effect on patient care and staff's workload/wellbeing, and possible barriers to implementation.

**Result:**

Comparing the two 3-month periods before and after SH implementation, restraint episodes were reduced from 47 to 21, seclusion episodes from 19 to 2, and rapid tranquilisation episodes from 10 to 3. Nine staff members responded to the feedback questionnaire. All believed SH had a positive impact on patient care, or had the potential to do so. Staff reported SH gave them insight into incidents, made them feel safer and prepared for the day, played a part in reducing restrictive practice, and empowered staff from all professional backgrounds by giving them a voice. Low or late participation, cancellation of SH because of clinical activity, and vague questions in the meeting template were identified as barriers in implementation.

**Conclusion:**

Acute psychiatric wards regularly face challenges of high clinical activity, low staffing levels, bed pressures, and high-risk patient cohorts. SH contributed to reducing restrictive practice and creating a safer and more positive work environment. It is important to ensure SH are taking place daily, using an appropriate template to guide staff who may be new to facilitating. Accordingly, the impact on restrictive practice, patient care and staff wellbeing can be sustained long-term.

